# Moist exposed burn ointment accelerates diabetes-related wound healing by promoting re-epithelialization

**DOI:** 10.3389/fmed.2022.1042015

**Published:** 2023-01-10

**Authors:** Yuanxun Gong, Yan Jiang, Jinmei Huang, Zuofen He, Qianli Tang

**Affiliations:** ^1^College of Integrated Traditional Chinese and Western Medicine, Hunan University of Chinese Medicine, Changsha, Hunan, China; ^2^Affiliated Hospital of Youjiang Medical University for Nationalities, Baise, China; ^3^West Guangxi Key Laboratory for Prevention and Treatment of High-Incidence Diseases, Youjiang Medical University for Nationalities, Baise, China; ^4^Graduate School, Guangxi University of Chinese Medicine, Nanning, China; ^5^Graduate School, YouJiang Medical University for Nationalities, Baise, China

**Keywords:** diabetic wound healing, re-epithelialization, moist exposed burn ointment, keratinocytes, cytokeratin

## Abstract

**Background:**

The incidence of diabetes-related wounds is widespread, and the treatment is challenging. We found that Moist Exposed Burn Ointment (MEBO) promotes the healing of diabetes-related wounds, but the mechanism is not clear.

**Methods:**

This study aimed to explore the mechanism of MEBO on diabetic wound healing, which may be related to the promotion of re-epithelialization. A full-thickness skin resection model was established in streptozotocin (STZ)-induced diabetic mice. MEBO and Kangfuxin (KFX) were applied to the wound area, and the wound healing rate was analyzed by photographing. The granulation tissue and epidermal thickness, the collagen remodeling rate, and the expression of cytokeratin 10 (CK10), cytokeratin 14 (CK14), Ki67, Collagen I, and Collagen III in the regenerated skin were detected by H&E staining, Masson staining, and immunofluorescence staining, respectively. MEBO and KFX were applied to human immortalized keratinocytes (HaCaT), mouse dermal fibrolasts (MDF) cells, and cell viability, cell migration, and differentiation were determined by CCK-8, scratching assay, RT-qPCR, and Western blot (WB), respectively.

**Results:**

We found that MEBO significantly promoted the formation of wound granulation tissue and collagen remodeling in diabetic mice. The application of MEBO to diabetic wounds not only promoted the formation of hair follicles and sebaceous glands but also promoted the expression of Ki67, CK10, and CK14 in epidermal cells. MEBO had no significant effect on the differentiation process of keratinocytes.

**Conclusion:**

Our study further proved that MEBO plays a positive role in diabetic wound healing, and its excellent ability to promote re-epithelialization may be an important reason for promoting wound healing.

## Background

The number of people living with diabetes is projected to increase by 25% in 2030 (1). As many as 30% of diabetic patients develop diabetic foot ulcers (DFU) during their lifetime (2), which is one of the important causes of disability and death of diabetes. DFU is characterized by a low cure rate, high recurrence rate, and high poor prognosis rate, and seriously affects the living quality and the lifetime of diabetic patients (3). Meanwhile, It also causes exceptionally heavy pressure on social, economic, and medical (4, 5).

The issues of wound healing and limb preservation in diabetes have attracted much attention over the past decade because of its potentially serious consequences (6). Researchers have focused on how to promote diabetic chronic wound healing by shortening wound closure time, enhancing the cost-effectiveness of treatment regimens, and improving limb retention (7, 8). In recent years, it has been recognized that optimal wound healing requires a combination of methods to solve a variety of clinical problems (9), such as persistent inflammation, insufficient angiogenesis, and impaired re-epithelialization.

Substantial studies have shown that the development of chronic refractory wounds is influenced by many factors (10). Generally, the process of wound repair can be divided into three stages: inflammation, proliferation, and remodeling (11). The proliferative stage is the most critical step in the process of wound repair, which involves the proliferation and migration of various cells, including endothelial cells, fibroblasts, keratinocytes, and macrophages. More specifically, the proliferation stage can be divided into two parts, re-epithelialization and granulation tissue formation, which complement each other, are closely linked, and affect each other (12). Generally, granulation tissue fills in the tissue defect immediately after injury, and the epidermis gradually covers the wound to complete the re-epithelialization process. Therefore, re-epithelialization is an important sign of wound healing. However, previous studies have shown that keratinocytes can not migrate and cover the wound in time, but form excessive hyperplasia epithelium at the edge of the wounds, which is one of the important reasons for the non-healing of diabetic wounds (13, 14).

Moist exposed burn ointment is a topical ointment for burn wounds and chronic refractory wounds, which has been widely used because of its remarkable clinical effect (15, 16). Therefore, some researchers have applied MEBO to diabetes-related chronic wounds and achieved good efficacy (17). In this study, we demonstrated that topical MEBO can induce wound re-epithelialization and collagen remodeling to promote wound healing in a full-thickness skin resection model in diabetic mice. Furthermore, to explore the mechanism of MEBO promoting diabetic chronic wound healing, we investigated the effects of MEBO on the proliferation, differentiation, and migration of human keratinocytes (HaCaT).

## Materials and methods

### Animals

All animal experimental protocols were approved by the Animal Care and Use Committee of Youjiang Medical University for Nationalities, Baise, China (2021030103). None of the authors are members of this committee. The care of animals was to the guidelines of the US National Institutes of Health (NIH) and the Chinese National Institute of Health. Male C57BL/6 mice (8 weeks old) were obtained from Changsha Tianqin Biotechnology Co., Ltd. (Changsha, China). The mice were housed in individually ventilated cages with pellet food and water. The animal housing rooms were maintained at a constant temperature of 20°C–24°C and humidity of 45%–65% with a 12-h light/dark cycle throughout the study. All surgical interventions were performed under anesthesia with 2.5% tribromoethanol (250 mg/kg body weight), using a standardized protocol established in our laboratory. All efforts were made to reduce the number of animals used and to minimize animal discomfort.

### Isolation and culture of MDF

Skin fibroblasts were isolated from the back skin of healthy newborn C57BL/6 mice. The pieces of skin were washed with PBS and digested with 0.01% collagenase type 1 with 0.1% Trypsin (Sigma-Aldrich; St. Louis, MO, USA) for 30∼60 min at 37°C. The digestion was terminated by the addition of a complete DMEM medium. Fibroblasts were collected by centrifugation and later cultured in a complete DMEM medium at 37°C in a 5% CO_2_ atmosphere before use.

### Cell culture

The HaCaT cells were purchased from the Cell Bank of the Chinese Academy of Sciences (Shanghai, China). HaCaT and MDF cells were grown in high-glucose Dulbecco’s modified Eagle’s medium (DMEM) containing 10% fetal bovine serum (FBS; Euroclone, Pero, Italy) and 1% penicillin and streptomycin (Gibco, Invitrogen Life Technologies, S. Giuliano Milanese, Italy). Cells were cultured at 37°C in a 5% CO_2_ atmosphere. The cell culture medium was changed twice per week. 0.1 g MEBO (Shantou MEBO Pharmaceutical Co., Ltd., China) was mixed with 1ml DMSO, assisted by ultrasonic dissolution, filtered, and debacterialized before dissolved in DMEM for subsequent cell experiments. Kangfuxing (Haoyisheng, Sichuan, China) is dissolved in DMEM before being used for subsequent cell experiments.

### Mouse wound model and treatment

The mice were randomly assigned to Non-diabetic and diabetic groups. To simulate human type 2 diabetes, the mouse diabetes wound model was established by high-fat diet + intraperitoneal injection of streptozocin (150 mg/kg body weight) once. Blood glucose levels measured at 4th, 5th and 6th day after STZ injection. The mice with fasting blood glucose of 16.7 mmol/L meanwhile with the symptoms of polyuria, polydipsia, and weight gain were considered a successful model of diabetes. The dorsal area of the mice was shaved, and an excisional wound 10 mm in diameter was made on the dorsal thorax with a Scalpel knife while the animals were under tribromoethanol anesthesia. Mice in the Non-diabetic group were treated by applying physiological saline to the wound. Mice in the Diabetic group were further randomly assigned to MEBO, KFX, and Diabetic groups and applied with MEBO (0.2 g/cm^2^), Kangfuxin liquid (0.2 ml/cm^2^), and physiological saline once a day for 3,7,10, and 14 days, respectively, with a cotton swab spreading from the center of the wound and spreading evenly around. Digital photographs of the healing wounds were captured at regular time points with a digital camera at an equal distance and a calibration scale on the side. Wound area closure was determined by ImageJ software. On day 3,7,10, and 14, mice in each group were euthanized by cervical dislocation, and skin specimens were harvested. The wound closure rate was calculated as Wound closure percentage (%) = [(area on day 0-area on day n)/area on day 0] × 100%.

### Measurement of transepidermal water loss in wounded area

Transepidermal water loss (TEWL) rates on the wounded areas of mice were measured with a TM300 probe, attached to a Courage-Khazaka MPA5 system (Courage & Khazaka, Cologne, Germany) under controlled atmospheric conditions (23°C, 60% relative humidity) at day 3, 7, 10 and 14 after wound. Measurements were performed by placing probes on the wounded area, locating this area in the middle. Measurements were taken before MEBO/KFX/physiological saline application.

### Histological analysis and immunofluorescence staining

After fixation with a 4% paraformaldehyde solution for 24 h, the samples were dehydrated, embedded in paraffin, and then sliced into 5 μm sections. Masson’s trichrome staining and hematoxylin and eosin (HE) staining were performed on the tissue slides. The tissue slides were imaged using an FSX100 microscope (Olympus, Tokyo, Japan). Three regions with the same area were selected randomly from each skin specimen to calculate the epidermal thickness, length of migrating epithelial tongue, and collagen deposition of each sample with ImageJ. Besides, the Immunofluorescence staining for cytokeratin 10 (CK10) and cytokeratin 14 (CK14), Ki67 were observed in the epidermal using fluorescence microscopy(Leica confocal STELLARIS 5).

### Cell viability measurement

The effect of MEBO and KFX on cell viability was measured by a CCK-8 assay (Dojindo, Japan). Briefly, HaCat/MDF cells were added to 96-well plates and cultured for 48 h. One group that was cultured in a DMEM medium was employed as a control. The supernatant fluid of the MEBO and the KFX group were replaced by MEBO+DMEM, and KFX+DMEM, there were six parallel samples for each group (100 μL of culture solution was added in each well). After 48 h, 10 μL CCK-8 reagent was added to the well and incubated for 1 h. The absorbance at 450 nm of each well was measured with a microplate reader (BioTek, VT, USA).

### Cell migration assay

For the scratch assay, HaCat and MDF cells were seeded in the 6-well plates respectively and cultured in DMEM medium until reaching 90% density. Cells were starved for 8–12 h, and mitomycin C (Sigma Aldrich, USA) was added into the medium by 15 μg/ml. Then cells were cultured in a 5% CO_2_ incubator at 37°C for 3 h, the mediums were replaced with a normally completed medium. The scratch was created in confluent monolayers of each cell by using a sterile P200 pipette tip and washed three times with PBS. Images of the samples were taken at 12 h and 24 h after scratching. The widths of the scratches were measured, recorded, and then compared with the original scratches at 0h using an inverted microscope and Image-Pro Plus software (Medical Cybernetics, USA). The area between the edges of the wound was measured with ImageJ software.

### Real-time quantitative polymerase chain reaction

Trizol reagent (Invitrogen) was used for total RNA isolation from mice skin tissues and cultured cells. cDNA was synthesized by FastKing gDNA Dispelling RT SuperMix (TIANJIN, China) and qRT-PCR was conducted by PreMixPlus SYBR Green (TIANJIN, China). Glyceraldehyde 3-phosphate dehydrogenase (GAPDH) and β-actin were used as internal standards. A Step One Real-Time PCR system (Life Technologies, Carlsbad, CA, USA) was used for RT-qPCR reactions. And the 2^–ΔΔ*Ct*^ approach was used for relative expression quantification. All primers are as follows: CK-10 5′—3′ F:ATGTCTGTTCGATACAGCTCAAG; R:CTCCACCAAGGGAGCCTTTG; CK-14 5′—3′ F:TGGG CAGTGAGAAGGTGAC; R:CAATGGTCTTGAAGTAGGGA CT; GAPDH 5′—3′ F:GGAAGCTTGTCATCAATGGAAA TC; R:TGATGACCCTTTTGGCTCCC; MMP9: 5′—3′ F: CTGGACAGCCAGACACTAAAG; R: CTCGCGGCAAGTCT TCAGAG; Collagen-I:5′—3′ F: GTAACTTCGTGCCTAGCA ACA; R: CCTTTGTCAGAATACTGAGCAGC; Collagen-III: 5′—3′ F: CTGTAACATGGAAACTGGGGAAA; R: CCATA GCTGAACTGAAAACCACC.

### Western blot analysis

Proteins were extracted by RIPA buffer (Invitrogen) and separated by 10% SDS-PAGE gels, transferred onto the Polyvinylidene Fluoride (PVDF) membrane, and probed with the appropriate primary antibodies specific for anti-cytokeratin 10 (CK10; Affinity), anti-cytokeratin 14 (CK14; Affinity),anti-Matrix metalloproteinase-9 (MMP9; Abcam), anti-Collagen-I(Abcam), anti-Collagen-III(Affinity) at 4°C overnight, and goat anti-mouse secondary antibody at 37°C for 50 min. The primary antibodies included Fluorescence scanning imaging. ImageJ software was used to analyze and process the strips.

### Statistical analysis

All data presented in this research came from at least three separate experiments and are reported as the mean ± standard deviation (SD). GraphPad Prism 8.0 software was used for statistical analysis. Two-way ANOVA was employed for comparison among groups at different time points. One-way ANOVA with Student–Newman–Keuls test was used for pairwise comparisons within a group.

## Results

### MEBO promoted diabetic wound healing in mice

**FIGURE 1:**
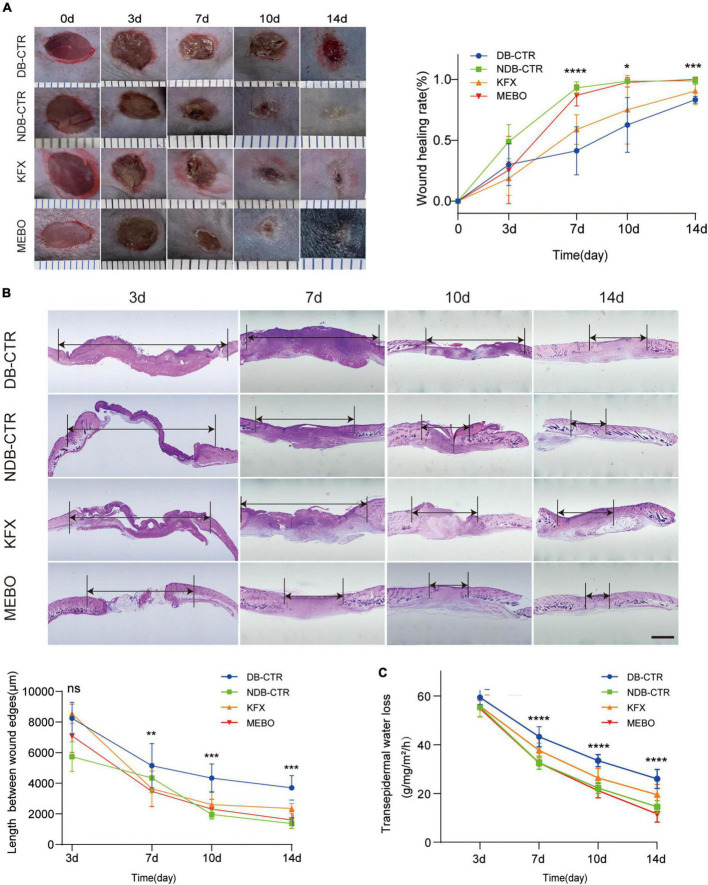
MEBO promoted diabetic wound healing in mice. **(A)** The representative images of the appearance of wound healing over time in each group. The scale is 1,000 μm. The wound healing rate of mice in each group was quantitatively analyzed by ImageJ software. **(B)** Representative images of HE staining of the wound skin at 3,7,10, and 14 day. The length between wound edges were measured by NIS-Elements Viewer. The scale is 1,000 μm. **(C)** Transepidermal water loss rates were measured with TM300. Diabetic Control (DB–CTR): Diabetic mice were treated with physiological saline on the wound surface. Non-diabetic Control (NDB–CTR): Normal mice were treated with physiological saline on the wound surface. KFX: Diabetic mice were treated with KFX on the wound surface. MEBO: Diabetic mice were treated with MEBO on the wound surface. At least 6 independent sections were chosen at each time for each group. All Data are shown as mean ± SD, ns *P* > 0.05, *P < 0.05, **P < 0.01, ***P < 0.001, ****P < 0.0001.

To study the effect of MEBO on diabetic wounds, STZ was used to induce diabetes in mice and a full-thickness skin resection model was established (18). Next, we applied MEBO as the treatment group in the wound area of diabetic mice. Meanwhile, We chose to use KFX as a positive control group, which is known to promote wound healing (19–21). During the treatment, we took photos at several time points to record the wound healing process of diabetic mice. We observed that there was no significant advantage in groups during the early healing period on the 3rd day. From the 7th day, compared with the diabetic control group and KFX group, the MEBO group showed a higher wound healing rate, which is comparable to the non-diabetic control group. On the 14th day, the wounds in the MEBO group had healed completely (Figure 1A).

Furthermore, the length of migratory epithelial tongue was measured by H&E staining in the regenerated skin tissue of the wound at day 3, 7, 10, and 14 post wounding. The results of H&E staining showed that MEBO effectively promoted wound healing in diabetic mice, which was basically consistent with the wound healing trend recorded by photographs (Figure 1B). These results show that MEBO has a stronger effect on promoting diabetic-associated wound healing than KFX.

When skin is injured, its barrier function is impaired, resulting in higher transepidermal water loss (TEWL), an index to assess skin barrier function *in vivo*. Therefore, in addition to visual documentation of wound closure, a TEWL assay was performed to quantitatively determine the re-establishment of skin barrier function following wounds. The results of TEWL assay showed that MEBO accelerated the recovery of barrier function in the wounds of diabetic mice compared with the diabetic control group (Figure 1C).

### MEBO accelerated collagen remodeling in diabetic wounds of mice

**FIGURE 2:**
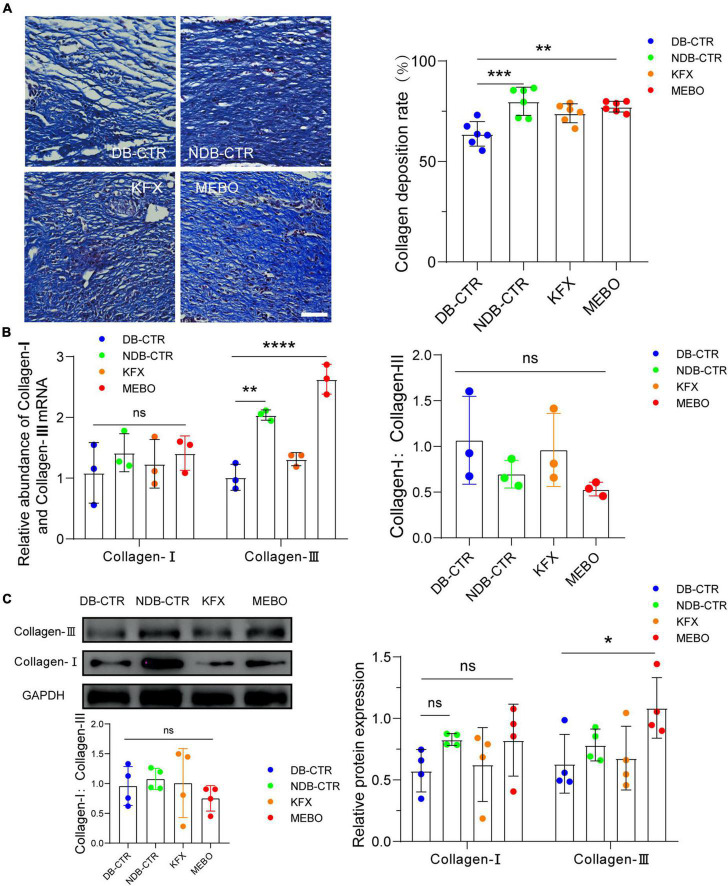
MEBO accelerated collagen remodeling in diabetic wounds of mice. **(A)** Representative images of Masson staining of regenerated skin tissue of mice in each group. The scale is 50 μm. The collagen remodeling of the regenerated skin of mice in each group was quantitatively analyzed by ImageJ software. **(B)** The mRNA relative contents of Collagen-I and Collagen-III of each group were compared by RT-qPCR. And the ratio of Collagen-I: Collagen-III were presented. **(C)** The protein expression of Collagen-I and Collagen-III of each group was determined by WB. And the ratio of Collagen-I: Collagen-III were presented. GAPDH serves as a loading control. The grayscale values of each strip were quantitatively analyzed by ImageJ software. At least 3 independent sections were chosen at each time for each group. All Data are shown as mean ± SD, ns *P* > 0.05, *P < 0.05, **P < 0.01, ***P < 0.001, ****P < 0.0001.

Collagen is a major component of the extracellular matrix. Diabetic wound healing is often accompanied by reduced collagen remodeling, which reduces the strength and elasticity of regenerated skin tissue and leads to more vulnerable wounds and insufficient scaffolds for cell attachment. Therefore, to evaluate the collagen remodeling level of the regenerated skin in each group, we performed Masson staining on wound tissue sections of each group on the 14th day after wounding. The results showed that the collagen remodeling rate of the MEBO group was higher than that of the Diabetic control group (Figure 2A). In addition, to confirm the promoting effect of MEBO on wound collagen remodeling, RT-qPCR and WB were applied to detect the mRNA and protein expression levels of collagen I and collagen III in the regenerated skin of mice in each group on day 14. The results showed that the expression level of collagen III in the MEBO group was significantly higher than that in the diabetic control group, while there was no significant difference in the expression level of collagen I among the groups (Figures 2B, C). But the ratio of collagen I:collagen III were not statistically significant in this study. These results suggest that application of MEBO to diabetic wounds promotes collagen remodeling in regenerating skin.

### MEBO accelerated the formation of granulation tissue and mature epithelial structure in diabetic wounds

**FIGURE 3:**
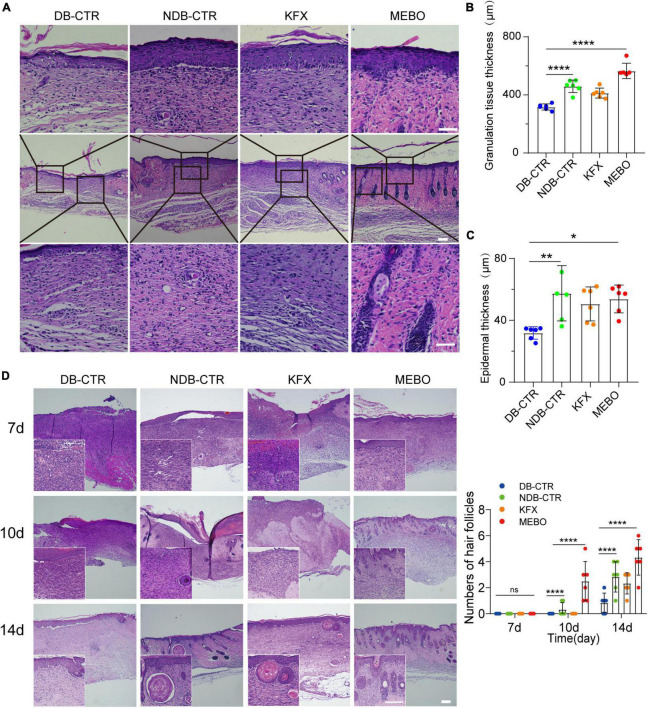
MEBO accelerated the formation of granulation tissue and mature epithelial structure in diabetic wounds. **(A)** The representative images of H&E staining of regenerated skin tissue of mice in each group on the 14th day after surgery. The scale is 50 μm. **(B)** The granulation tissue thickness of regenerated skin in each group was quantitatively analyzed by ImageJ software. **(C)** The epidermis thickness of regenerated skin in each group was quantitatively analyzed by ImageJ software. **(D)** High-magnification images showing the formation process of hair follicles in each group at days 7, 10, and 14. The scale is 500 μm (10X) and 50 μm (40X). The number of hair follicles in each group was calculated. At least 6 independent sections were chosen at each time for each group. All Data are shown as mean ± SD, ns *P* > 0.05, *P < 0.05, **P < 0.01, ***P < 0.001, ****P < 0.0001.

To further understand the effect of MEBO on diabetic wounds, we performed H&E staining and histological evaluation of the wound tissue on the 14th day after surgery. The results showed that the regenerated skin tissues in the MEBO group were more epithelialized than those in the other three groups (Figure 3A). To quantitatively compare the differences in the degree of re-epithelialization among each group, we measured the thickness of granulation tissue and epidermis at the wound surface of each group and statistically analyzed the results. The results showed that the thickness of granulation tissue of the MEBO group was higher than that in the diabetic control group (Figure 3B). Similarly, quantitative statistical results also showed that the epidermal thickness of the MEBO group was higher than that of the diabetic control group (Figure 3C).

Furthermore, we focused on the H&E stained images of wound tissues at day 7, 10, and 14. Unexpectedly, functional structures such as hair follicles and sebaceous glands were first developed in the regenerated skin of the MEBO group at day 10 and matured at day 14, indicating that the regenerated skin of the MEBO group already had basic functional components (Figure 3D). These results suggest that MEBO promoted diabetic wound healing and accelerated the formation of mature epithelial structures.

### MEBO promoted re-epithelialization in the process of diabetic wound healing

**FIGURE 4:**
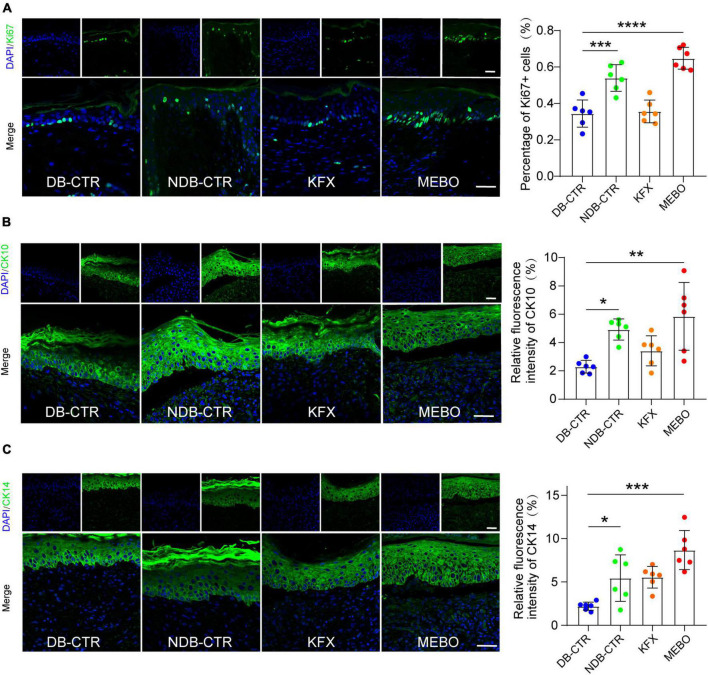
MEBO promoted re-epithelialization in the process of diabetic wound healing. **(A)** The representative immunofluorescence images of regenerated skin tissue of mice in each group were taken by a confocal microscope, the green was Ki67, DAPI, and the nucleus were blue. The percentage of Ki67-positive basal layer cells in the regenerated epidermis of mice in each group was statistically analyzed by ImageJ software. **(B)** The representative immunofluorescence images of regenerated skin tissue of mice in each group were taken by a confocal microscope, the green was CK10, DAPI, and the nucleus were blue. The percentage of CK10-positive cells in the regenerated epidermis of mice in each group was statistically analyzed by ImageJ software. **(C)** The representative immunofluorescence images of regenerated skin tissue of mice in each group were taken by a confocal microscope, the green was CK14, DAPI, and the nucleus were blue. The percentage of CK14-positive cells in the regenerated epidermis of mice in each group was statistically analyzed by ImageJ software. The scale was 50 μm. At least 6 independent sections were chosen at each time for each group. All Data are shown as mean i± SD, *P < 0.05, **P < 0.01, ***P < 0.001, ****P < 0.0001.

To understand the effects of MEBO on the re-epithelialization of diabetic wounds, we performed immunofluorescence analysis on the regenerated skin tissue sections of each group. Ki67 is a classic proliferative intranuclear marker (22). Immunofluorescence results showed that the positive rate of Ki67 in basal layer cells of the regenerated epidermis in the MEBO group was significantly higher than that in the diabetic control group and KFX group, which suggested the positive effect of MEBO on re-epithelialization of diabetic wounds (Figure 4A). CK10 is often expressed in mature epidermal basal layer cells, which are a marker of mature epidermal cells (23). CK14 is a proliferation marker of epidermal basal layer cells (24). Due to the stable distribution and expression of keratins, we detected the expression of CK10 and CK14 in the regenerated skin of mice in each group by immunofluorescence staining. The results showed that the expression of CK10 and CK14 in the regenerated epidermis of the MEBO group were significantly higher than those of the diabetic control group (Figure 4B, C). These results indicated that the maturity of epidermal differentiation in the MEBO group was higher than that in other groups, suggesting that MEBO had an excellent ability to promote re-epithelialization.

### MEBO accelerates the process of re-epithelialization by promoting the migration of keratinocytes

**FIGURE 5:**
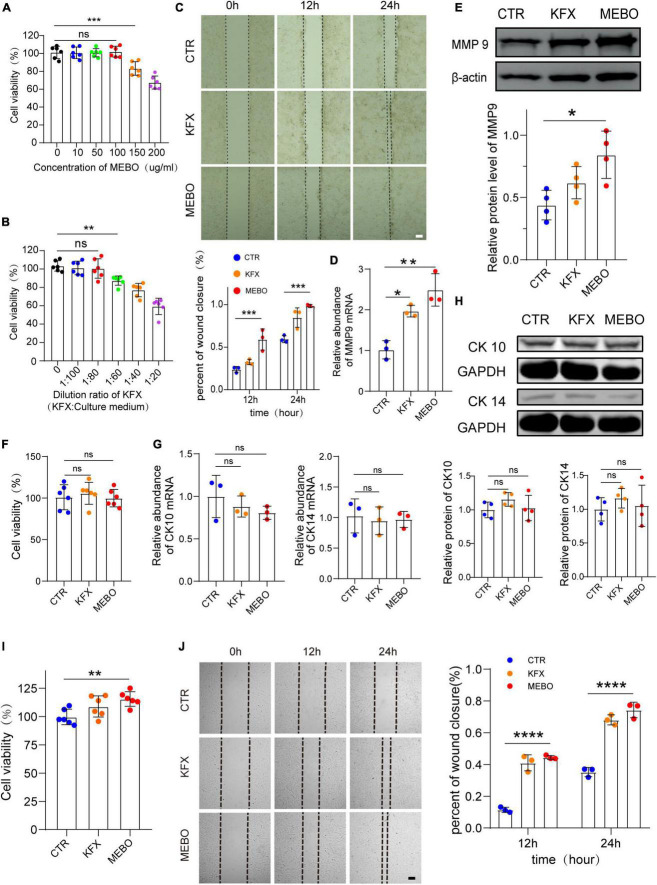
MEBO accelerates the process of re-epithelialization by promoting the migration of keratinocytes. **(A)** The cell viability of different concentration of MEBO applied on HaCaT cells was measured by cell counting kit-8 assay. **(B)** The cell viability of different ratio of KFX applied on HaCaT cells were measured by cell counting kit-8 assay. **(C)** The representative images of wound closure of HaCaT cells at different time points in each group were taken by an inverted microscope. The scale was 100 μm. The Percentage of wound closure of HaCaT cells at different time points in each group was statistically analyzed by ImageJ software. **(D)** The mRNA relative contents of MMP9 in HaCaT cells of each group were compared by RT-qPCR. **(E)** The protein expression of MMP9 in HaCaT cells of each group was determined by WB. β-actin serves as a loading control. The grayscale values of each strip were quantitatively analyzed by ImageJ software. **(F)** The cell viability of HaCaT cells in each group was determined by cell counting kit-8 assay. **(G)** The mRNA relative contents of CK10 and CK14 in HaCaT cells of each group were compared by RT-qPCR. **(H)** The protein expression of CK10 and CK14 in HaCaT cells of each group was determined by WB. GAPDH serves as a loading control. **(I)** The cell viability of MDF cells in each group was determined by cell counting kit-8 assay. **(J)** The representative images of wound closure of MDF cells at different time points in each group were taken by an inverted microscope. The scale was 100 μm. The Percentage of wound closure of MDF cells at different time points in each group was statistically analyzed by ImageJ software. The scale was 100 μm. All Data are shown as mean ± SD, *N* ≥ 3, ns *P* > 0.05, *P < 0.05, **P < 0.01, ***P < 0.001, ****P < 0.0001.

The efficiency of wound healing depends upon the migration rate of the keratinocytes. Non-migrating hyperproliferative keratinocytes are characteristic of non-healing diabetic wounds. Therefore, the effect of MEBO on HaCaT cell migration was evaluated by scratch assay. Similarly, to objectively evaluate the effect of MEBO on HaCaT migration, we selected KFX as a positive control. CCK-8 assay was applied to evaluate the optimal concentration of MEBO and KFX *in vitro*. According to the results of CCK-8 assay, MEBO solution at a concentration of 100ug/ml and KFX solution diluted 80 times were applied to the experiments *in vitro* (Figures 5A, B). Upon MEBO treatment in serum-free media, faster wound closure of HaCaT cells as compared to other group cells was observed. Complete wound closure occurred in MEBO-treated HaCaT cells within 24 h, whereas the wound was still open in control group cells (Figure 5C). Furthermore, the mRNA and protein levels of MMP9 were detected by RT-qPCR and WB, and the results showed that the expression of MMP9 was up-regulated in HaCaT cells after MEBO treatment (Figures 5D, E). The results suggested that MEBO can promote the migration of HaCaT cells.

Next, to avoid MEBO-induced proliferation during wound closure, we evaluated the effect of MEBO on HaCaT cell proliferation through CCK-8 colorimetry. The results showed that there was no significant difference in cell viability among the three groups after 48 hours of treatment (Figure 5F). Therefore, it was confirmed that the migration accelerated by MEBO is not caused by cell proliferation. Furthermore, to confirm whether the results of accelerated migration were related to the differentiation of HaCaT cells, the mRNA expression levels of CK10 and CK14 in each group were detected by RT-qPCR and the protein expression levels of CK10 and CK14 were detected by WB after 48 h after treatment. There was no significant change in the expression of CK10 and CK14 in HaCaT cells in the MEBO group and the other groups, which suggested that MEBO had no significant effect on the differentiation process of keratinocytes (Figures 5G, H). Taken together, we concluded that MEBO accelerated re-epithelialization by promoting keratinocyte migration.

Since the producing and thickness of collagen is related to the proliferation and migration of fibroblasts, we investigated the effects of MEBO on MDF. The results showed that MEBO significantly enhanced the proliferation and migration ability of MDF, which may partially explain the mechanism of MEBO promoting collagen remodeling in the wounds of diabetic mice (Figures 5I, J).

## Discussion

Although many studies have shown the effect of MEBO in the treatment of burns and chronic wounds (25–27), its mechanism is still unclear. In this study, we found that MEBO significantly promoted wound healing in diabetic mice. Histological results showed that the collagen remodeling rate of regenerated skin in the MEBO group was higher than that in the diabetic control group. Meanwhile, hair follicles and sebaceous glands were first formed at the wound site in the MEBO group, indicating that the wound of the MEBO group was the first to complete the re-epithelialization process. The epidermal proliferation and differentiation markers such as Ki67, CK10, and CK14 detected by immunofluorescence verified the accelerating effect of MEBO on re-epithelialization. Furthermore, *in vitro* results suggested that the effect of MEBO on re-epithelialization may depend on the promotion of keratinocyte migration rather than proliferation and differentiation and the promotion of collagen regeneration by MEBO may be related to the mobilization of dermal fibroblasts. To sum up, our findings confirm the role of MEBO in promoting diabetic wound healing and provide a new theoretical basis for the clinical application of MEBO in the treatment of diabetic-associated chronic wounds.

Due to the complexity of diabetic wounds, the clinical treatment of chronic diabetic wounds often requires the combined application of multiple methods (28, 29). In recent years, some new technologies for diabetic wounds have been gradually developed, such as surgery or stem cell transplantation, to achieve the best efficacy (30, 31). However, some patients can only take conservative treatment due to their advanced age or multiple underlying diseases, resulting in poor treatment results. Therefore, the traditional treatment plan still deserves our attention. In its long-term practice, traditional Chinese medicine has accumulated many rich and effective methods to treat chronic wounds (32, 33).

Moist exposed burn ointment is an external Chinese medicine agent, which is easy to apply, regardless of the size, scope, and local conditions of the wound (27). It is an oil-based ointment that contains many natural herbs and plant ingredients. It provides physiological moisture necessary for wound healing and re-epithelialization. Moreover, it also has the pharmacological effect of preventing skin dehydration, anti-inflammation, antibacterial, and analgesia (34, 35). After nearly 30 years of research and clinical application, the original moist exposure burn therapy has gradually matured and developed into a medical technology of skin regeneration, which has been widely used in the treatment of chronic refractory wounds on the body surface. MEBO is not only widely recognized and used in China, but also introduced in many other countries, such as Thailand, Syria, South Korea, Singapore, the United Arab Emirates, etc (36). Although many clinical applications have proven the positive effect of MEBO on a burn and chronic wound healing, there are few basic studies on MEBO and its mechanism is still unclear, which is one of the important reasons hindering its further clinical promotion.

The epidermis is the outermost layer of the skin, and keratinocytes are the main cells in the epidermis. When an injury is occurring on the skin, keratinocytes migrate to the wound area, re-epithelializing the damaged tissue and restoring the epidermal barrier (37, 38). However, the important feature of non-healing diabetic wounds is excessive proliferation and non-migration of keratinocytes, resulting in continuous thickening of the wound edge epidermis, while the wound is unhealing (39). The key to scar-free embryonic (40) wound healing and complete healing of animals with high regenerative potential, such as salamanders, depends on rapid re-epithelialization (41). Furthermore, the changes in extracellular matrix (ECM) components in unhealed wounds and the increase in ECM degradation rate caused by increased levels of matrix metalloproteinases may damage the attachment of keratinocytes and lead to abnormal cell signals and migration disorders (42–45). Therefore, it is a promising therapeutic target for diabetic wounds to improve the migration barrier of keratinocytes to promote wound re-epithelialization (46). In the process of wound healing, tissue regeneration is a more desirable outcome than scar formation. Although fibrosis is more common after a skin injury, full skin regeneration leads to the complete replacement of appendages and function. Previous studies (47) have explained the regeneration of new hair follicles by activation of Wnt signaling after full-thickness injury in mice. Stem cell populations expressing hair follicle differentiation markers were established around these regenerated hair follicles and successfully transitioned through all stages of the hair cycle and further formed related structures such as sebaceous glands. However, what triggers skin appendage regeneration in mammals is not fully understood. In this research, we proved that MEBO can rapidly remodel collagen in regenerated skin, form granulation tissue, and re-epithelialize the wound by enhancing the migration ability of keratinocytes, which may be one of the important reasons why MEBO promotes wound healing. Meanwhile, functional structures such as hair follicles and sebaceous glands first formed in the regenerated skin of mice in the MEBO group are also of great significance for wound repair.

## Conclusion

In conclusion, our study further proved that MEBO plays a positive role in diabetic wound healing, and its excellent ability to promote re-epithelialization may be an important reason for promoting wound healing. Combined with previous studies, we believe that MEBO can accelerate the accumulation of collagen in regenerated skin by mobilizing dermal fibroblasts and promoting the migration of keratinocytes based on preventing skin dehydration, anti-inflammation, antibacterial, and analgesia, ultimately leading to wound healing. Moreover, the healed wound has a relatively intact skin appendage. This finding further strengthens our understanding of the role of MEBO in promoting chronic wound healing and provides new theoretical support for its further clinical promotion.

## Data availability statement

The original contributions presented in this study are included in this article/Supplementary material, further inquiries can be directed to the corresponding author.

## Ethics statement

The animal study was reviewed and approved by Animal Care and Use Committee of Youjiang Medical University for Nationalities, Baise, China (2021030103).

## Author contributions

YG conceived the project, performed most of the experiments, and wrote the manuscript. YJ performed parts of the experiments, data acquisition, and data analysis. JH and ZH prepared reagents and collected samples. QT designed the experiments and supervised the study. All authors read and approved the final manuscript.
