# Supplementary material for: Moist exposed burn ointment accelerates diabetes-related wound healing by promoting re-epithelialization
Source: Front Med (Lausanne). 2023 Jan 10;9:1042015. doi: 10.3389/fmed.2022.1042015 (PMC9871640; doi:10.3389/fmed.2022.1042015)
Supplement: Supplementary file 1 [file Table_1.DOCX]

https://www.jianguoyun.com/p/DbidU_4Qvsj5Chjgq9kEIAA
